# Effects of Electronic Cigarettes on Periodontal Health: A Systematic Review and Meta-Analysis

**DOI:** 10.1016/j.identj.2024.12.036

**Published:** 2025-01-24

**Authors:** Reem Alkattan, Nada Tashkandi, Amani Mirdad, Hossam Tharwat Ali, Nouf Alshibani, Eman Allam

**Affiliations:** a Department of Periodontics and Community Dentistry, King Saud University, Riyadh, Saudi Arabia; b Preventive Dentistry Department, Riyadh Elm University, Riyadh, Saudi Arabia; c Qena Faculty of Medicine, South Valley University, Qena, Egypt; d Research and Graduate Studies Department, Mohammed Bin Rashin University of Medicine and Health Sciences, Dubai, UAE

**Keywords:** Smoking, Cigarette, Vaping, Periodontal health, Periodontitis

## Abstract

**Objectives:**

The use of electronic cigarettes “e-cigarettes,” or vaping is growing in popularity, especially among adolescents and young adults. While the effects of cigarette smoking on oral health are well-established, the exact impact that e-cigarettes may have on dental tissues is still uncertain. The aim of the current review was to summarize evidence related to the effect of vaping on the periodontal health status of e-cigarette users.

**Methods:**

A comprehensive electronic search was performed using PubMed, Web of Science, and Scopus databases, until January 31^st^, 2024. Two independent reviewers participated in the screening of studies, data extraction, and assessment of the included studies. Any disagreements were resolved by a third reviewer the quality assessment was done using the Newcastle-Ottawa Scale to assess the risk of bias. A frequentist meta-analysis was performed using R Statistical Software. The random effects model was adopted. Data were described as mean difference (MD) and 95% confidence interval (CI). A p-value of ≤ .05 was deemed statistically significant.

**Results:**

Ten studies met the eligibility criteria. Overall, the findings were consistent, with most studies showing that e-cigarette users are at greater risk of periodontal disease than nonsmokers, but that they have a lower risk than cigarette smokers. Pooling results showed lower mean probing depth (PD) among nonsmokers than e-smokers (MD: -1.91; 95% CI: [-3.36: -0.47]; p-value = .01) while it was higher among cigarette smokers in participants with periodontitis (MD:0.43; 95%CI: [0.08:0.79]; p-value = .02). Compared to e-smoking, nonsmokers had lower PI (MD: -20.63; 95%CI: [-28.04: -13.21]; p-value < .001) while cigarette smokers had higher PI (MD: 4.88; 95% CI: [-1.52:11.29]; p-value = .135). Among participants with periodontitis, only cigarette smokers had significantly higher PI (MD: 4.53; 95%CI: [1.94:7.13]; p-value < .001).

**Conclusion:**

Based on the current analysis, conventional cigarette smoking is the most detrimental to periodontal health among the groups compared in all included studies. This indicates that traditional cigarettes have a more severe impact on periodontal tissues than do e-cigarettes. The data suggest a gradient of risk where nonsmokers have the lowest risk, e-cigarette users have a moderate risk, and cigarette smokers have the highest risk for periodontal health issues.

## Introduction

Since being introduced in 2004, vaping or e-cigarettes has seen a striking worldwide increased popularity, especially among teens and young adults.^1^ Vaping can be defined as inhaling an aerosolized “e-liquid”, produced by an electronic vaporization device, which does not require combustion. Instead of burning tobacco, as with traditional cigarettes, e-cigarettes heat up and vaporize nicotine and other flavoring products. Because of containing fewer ingredients and the absence of combustion, e-cigarettes and vaping products were, for a while, considered a safer alternative to cigarettes and even a potential tobacco-cessation product.^2^^,^^3^

An e-cigarette device contains a battery, a reservoir holding liquid, and a vaporization chamber with a heating element. The liquid is a solvent comprising nicotine, propylene glycol, glycerine, flavoring additives, and sweeteners. While several of the e-liquid formulations contain mostly nicotine, they can also contain other drugs such as tetrahydrocannabinol and other substances such as methamphetamine, methadone, and vitamins. When this liquid is heated, the e-cigarette creates an aerosol of fine particles that is systemically absorbed through the oral tissues and lungs for the nicotine to be delivered to the brain within a couple of seconds.^4^^,^^5^

Tobacco smoking is a major risk factor for several oral conditions including periodontal diseases and oral cancer. Cigarette smoke contains at least 500 potentially toxic substances, including hydrogen cyanide, carbon monoxide, free radicals, tar, nitrosamines, nicotine, and several oxidant gases. The periodontal effects of tobacco smoke are thought to occur via the systemic effects of these toxic constituents on immune function and the inflammatory response within the periodontal tissues. Nicotine, the main psychoactive, chemically addictive component in tobacco smoke, is a primary ingredient in e-cigarette liquids. Vaping, or the use of e-cigarettes, has been the subject of increasing scientific scrutiny regarding its potential impact on systemic and oral health. Several studies have attempted to discuss the impact of vaping on oral health and dentition.^6^, ^7^, ^8^, ^9^, ^10^

In vitro studies have demonstrated that aldehydes and free radicals contained in the aerosols of e-cigarettes cause oxidative stress, alterations in cellular antioxidant activity and DNA damage, changes that are expected to ultimately lead to periodontal tissue destruction and alveolar bone loss, characteristics of periodontal diseases. Some in vivo studies and clinical trials, however, had conflicting findings.^8^, ^9^, ^10^ Some reports have suggested that e-cigarettes are less harmful than conventional cigarettes but might still affect periodontal health.^11^, ^12^, ^13^ Other studies had reported significant oral health implications including vaping-related cancerous changes, more infections, xerostomia, and traumatic injuries or burns.^14^^,^^15^ The aim of the current study was to systematically review the available evidence on the effects of vaping on periodontal disease/periodontitis.

## Methods

### Protocol and registration

The present systematic review and meta-analysis was performed according to the Preferred Reporting Items for Systematic Reviews and Meta-analyses (PRISMA) statement.^16^^,^^17^ The review protocol was registered in the Prospective Register of Systematic Reviews (PROSPERO: CRD42024507375).

### Literature search

A systematic search of 3 electronic databases (PubMed, Web of Science, and Scopus) was conducted. The search included all possible terms that refer to vaping and periodontal health conditions (Supplementary data; Table S1). The search was restricted to English language and publication from January 2015 until January 31^st^, 2024. The published reviews and reference lists of selected papers were also searched.

### Eligibility criteria

The review employed the Population, Intervention, Comparison, Outcome, and Study Type (PICOS) framework. The study population consisted of individuals using vape or e-cigarettes, the intervention was defined as the use of vape or e-cigarettes, and the comparison was defined as nonsmokers or those smoking conventional cigarettes. The study intended to examine the association between e-cigarette use and periodontal health and thus the outcome was determined to be any changes to the periodontium that are either self-reported by the patients or confirmed through laboratory or clinical studies. Included study types were any published observational research reporting on the research question excluding in vitro and animal studies, case reports, editorials, conference papers, and reviews.

### Screening of the studies

The duplicate records were eliminated after the retrieval of citations. Two reviewers independently screened titles and abstracts of the citations retrieved by the literature search against the inclusion and exclusion criteria followed by a full-text assessment to confirm the study's selection decisions. A third reviewer was consulted to resolve any conflicts.

### Data extraction and outcome measures

Included studies underwent data extraction using specifically designed extraction forms. Two independent reviewers extracted data; a third independent reviewer resolved any differences. Extracted data included study methodology and design, participants' characteristics, and major findings. Outcome measures included periodontal parameters involving plaque index (PI), bleeding on probing (BOP), probing depth (PD), clinical attachment loss (CAL), marginal bone loss (MBL), and inflammatory mediators such as interleukins (ILs) and matrix metalloproteinases (MMPs) (Table 1).^18^, ^19^, ^20^, ^21^, ^22^, ^23^, ^24^, ^25^, ^26^, ^27^

**Table 1:** Summary of the included studies that evaluated clinical aspects of conventional or e-smoking.

Study	Study Design	Study Site	Sample Size	Male Percentage	Participants’ Age	Study Population	Study Arms			Follow-up Period (Months)	Outcome Measures	Major Study Findings
							E-smoking (Vaping)	Conventional smoking	No Smoking			
Akram et al.,^18^	Longitudinal study	Australia	60	100%	35.7 ± 14.5	Healthy/Periodontitis (split-mouth)	30	30	0	6	MMP-8, CTX, Plaque scores, BOP, PD, AL, MBL	Increased attachment loss, PD, MBL in diseases sites of cigarette smoking than e-cig at 6-month. Increased BOP among e-cig users compared with CS in periodontitis sites.
Al-Hamoudi et al.,^19^	Longitudinal study	Saudi Arabia	71	87%	NR	Chronic periodontitis	36	0	35	3	PI, GI, PD, AL, MBL, IL-4, IL-10, IL-11, IL-13.	Levels of GCF IL-4, IL-9, IL-10, and IL-13 increased after SRP in e-cig users and NS with CP; however, the anti-inflammatory effect of SRP was more profound in NS than in e-cig users.
Alharthi et al.,^20^	Prospective Cohort	Pakistan	89	100%	Range: 32.5-39.4	Gingival inflammation; All individuals with BOP in at least 30% of the sites.	28	30	31	3 and 6	PI, BOP, AL, PD, MT	Following FMUS, gingival inflammation is worse in CS compared with individuals vaping E-cigs and NS.
Ali et al.,^21^	Cross-sectional	Kuwait	75	72%	Range: 48.1- 58.7	Population with/without periodontitis	18	19	38	NA	PI, GI, AL, PD, MT, IL-15, IL-18,	Scores of PI, CAL, PD, MT and missing teeth were comparable among all smoking groups. IL-15 and IL-18 were elevated in smokers and vapers compared to NS.
ArRejaie et al.,^22^	Cross-sectional	Saudi Arabia	95	100%	Range: 35.8-45.3	Patients with at least one dental implant in all groups that has been in service for ≥36 months	31	32	32	NA	PI, BOP, PD, MBL, MMP-9, IL-1β	PI, PD, mean concentrations of MMP-9 and IL-1β were significantly higher in CS and e-cigs than NS. MBL was significantly higher in CS than NS and e-cigs users.
Shah et al.,^23^	Retrospective Cohort	UK	160	41%	Range: 18-77	Periodontitis	20	20	120	NR	PD, CAL, BOP, recession, plaque scores.	E-cig users had a statistically significantly less favourable response to PMPR than NS.
AlJasser et al.,^24^	Prospective Cohort	Saudi Arabia	60	52%	Range: 18–70 years	Peri-implant disease; Patients underwent at least 1 implant surgery during the past 2 years.	20	20	20	1, 6, 12 months	Gingival consistency, color, BOP, PI, PD, IL-1 β, IL-6, MMP-8, TNF- α and TIMP-1	Vapers had compromised response of peri-implantitis treatment.
Alqahtani et al.,^25^	Cross-sectional	Saudi Arabia	102	100%	Range: 32.2-37.5	Partially edentulous adults rehabilitated with dental implant	34	35	35	NR	Peri implant PI, PD, BOP	Scores of peri-implant PI and PD were significantly higher among CS, and e-cig users compared with NS.
BinShabaib et al.,^26^	Cross-sectional study	Saudi Arabia	135	92%	Range: 36.5-47.7	Normal population	44	46	45	NA	PI, BOP, PD, CAL, MBL, IL-1β, IL-6, tumor-necrosis-factor-alpha (TNF-α), MMP)-8, interferon-gamma (IFN-γ)	Periodontal status was poorer (PI, PD, CAL) and GCF levels of proinflammatory cytokines (IL-1β, IL-6, IFN-γ, TNF-α and MMP-8) were higher in CSs compared with e-cig smokers and NS.
Ibraheem et al.,^27^	Cross-sectional	Saudi Arabia	120	100%	Range: 43.8-51.8	Normal population	30	30	30	NA	PI, BOP, PD, CAL, RANKL, OPG	PI, PD, GCF RANKL and OPG levels were significantly higher among smokers, and e-cig users than NS.

BOP, bleeding on probing; PD, probing depth; MBL, marginal bone loss; PI, plaque index; CAL, clinical attachment loss; GI, gingival index; GCF, gingival crevicular fluid; IL, interleukin; MT, missing teeth; FMUS, full-mouth ultrasonic scaling; CTX , C-terminal crosslinked telopeptide of type I collagen; SRP, scaling and root planning; CP, chronic periodontitis; ENDS, electronic nicotine delivery systems; PMPR, professional mechanical plaque removal; UPR, unfolded protein response; NHOKs, normal human oral keratinocytes; 8-OHdG, 8-hydroxydeoxyguanosine; GSH-Px, Glutathione peroxidase; HGFs, human gingival fibroblasts; ROS, reactive oxygen species; NR, not able to retrieve; CS, cigarette smoker; NS, non-smoker; NA, not applicable.

PI was determined visually in all studies using either the Visible Plaque Index (VPI) method scoring by means of simple categorical definitions (presence or absence of plaque) or using the O´leary Index (DPI) where teeth are stained with a disclosing solution to detect the presence of plaque scored as a dichotomous variable. The final score per individual is then calculated as the sum of the plaque scores divided by the number of surfaces examined. CAL was assessed as measurements made from the cement–enamel junction (CEJ) to the base of the periodontal pocket while PD as measurements made form the free gingival margin to the base of the periodontal pocket. Bleeding on probing was assessed clinically (presence of bleeding -1, absence of bleeding – 0) while MBL was assessed radiographically and defined as the vertical distance from 2 mm below the CEJ to the alveolar crest.^18^^,^^20^^,^^21^

### Quality assessment of the studies

Two reviewers independently employed the Newcastle-Ottawa Scale (NOS) to assess the risk of bias in the included studies.^28^ The NOS is 1 of the most used tools for evaluating quality in meta-analyses of observational studies with confirmed validity and adaptability.^29^ For cohort studies, a study was awarded a maximum of 1 star for each numbered item within the selection and outcome categories. A maximum of 2 stars was given for comparability. For cross-sectional or case-control studies, a study was awarded a maximum of 1 star for each numbered item within the selection and exposure categories and a maximum of 2 stars was given for comparability. Studies were rated according to the total star score as follows: low-quality (< 4 stars), medium-quality (4-6 stars) and high-quality (≥ 7 stars).^28^ Assessment results of the included studies are shown in supplementary data (Table S2).

### Statistical analysis

The data were organized in a Microsoft Excel sheet and then imported and analyzed using R Statistical Software, {nutmeat} package (v4.1.3; R Core Team 2022).^30^ A frequentist network meta-analysis (NMA) comparing electronic cigarette smoking to both cigarette smoking and no-smoking was conducted. The study population was divided according to the primary studies into healthy sample (healthy subjects/sides with no periodontal diseases), periodontitis (patients diagnosed with periodontitis), and peri-implant diseases (patients with a history of dental implants and peri-implantitis). The random effects model was adopted due to the inherited differences within and between studies, populations, and measurements. Data were synthesized as mean difference (MD) and the corresponding 95% confidence interval (CI). A p-value of ≤ .05 was deemed statistically significant. The heterogeneity in the data was examined through I^2^ tests, following the Cochrane Handbook (chapter 9) for interpretations as follows: not significant for 0-40%, moderate heterogeneity for 30-60%, substantial heterogeneity for 50-90%, and considerable heterogeneity for 75-100%.^28^ Groups (smoking status) were ranked using the P-score which assesses the probability that a type is better than other options.^31^ Network plots for each outcome demonstrating the sample size and number of studies for each group were constructed.

## Results

### Search results

**Fig. 1:**
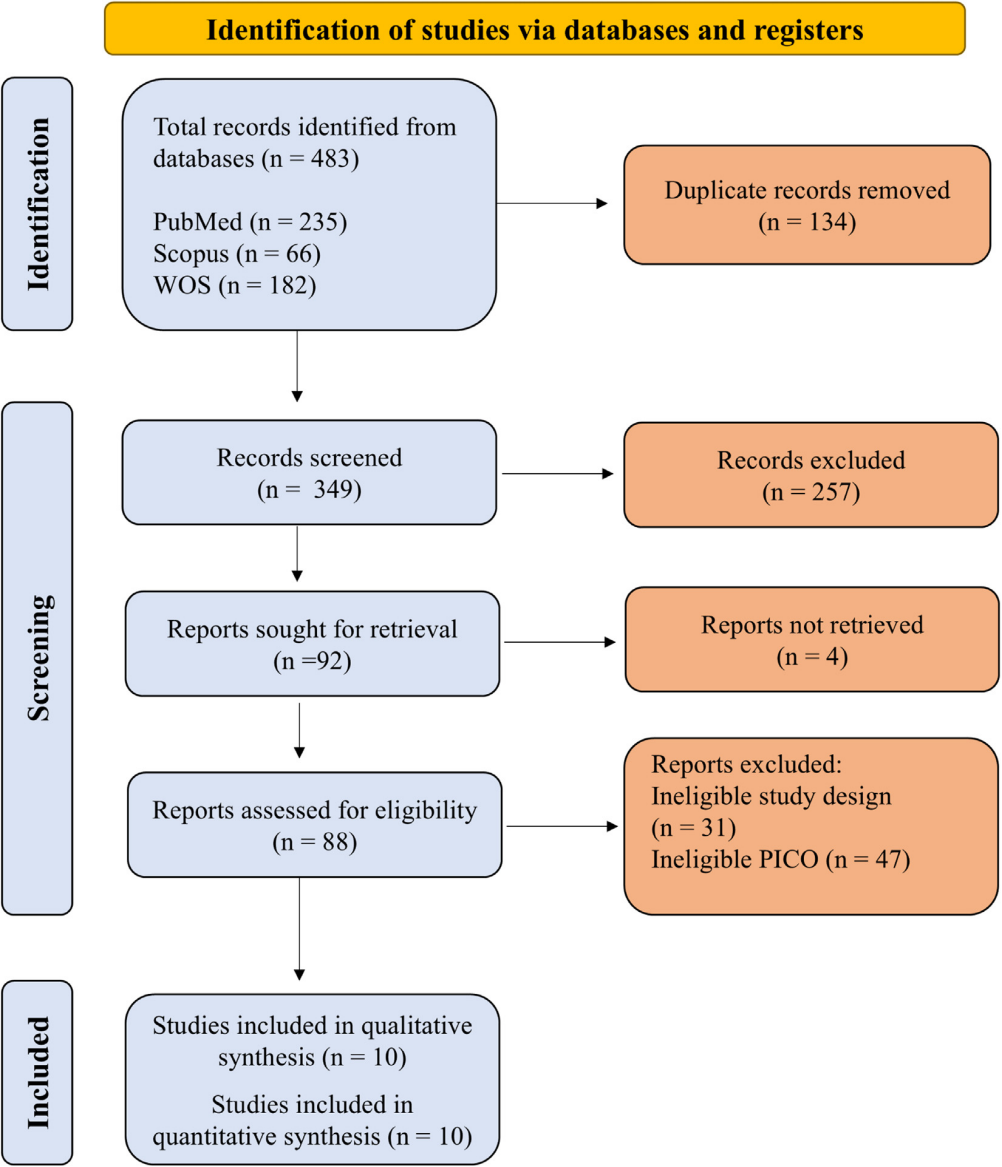
Summary of the search and process of the study selection.

A total of 483 records resulted from searching literature databases. After removing 134 duplicate records, we screened the title and abstract of 349 references. Having excluded 257 records, we sought retrieval of 92 articles for full-text review. Four reports could not be retrieved. Of the 88 reports assessed for eligibility, 31 were of ineligible study design and 47 had ineligible PICO elements. Ten reports met our inclusion criteria and were finally included in our review (Figure 1).

### Studies’ characteristics and qualitative synthesis

The characteristics of the studies included in this review are summarized in Table 1.^18^, ^19^, ^20^, ^21^, ^22^, ^23^, ^24^, ^25^, ^26^, ^27^ The studies were conducted between 2019 and 2024, a span of 5 years. Most of these studies had been conducted in Saudi Arabia (n=6), with others conducted in Pakistan (n=1), Kuwait (n=1), Australia (1), and the United Kingdom (n=1). Their primary objective was to assess the effect of electronic cigarettes on periodontal health. Most compared periodontal parameters (clinical indicators and inflammatory markers) between vapers, cigarette smokers, and non-smokers.

Some studies^20^^,^^23^^,^^24^ investigated the effect of vaping on treatment response while others^20^^,^^22^^,^^23^ investigated the effects of vaping on peri-implantitis. In addition to the periodontal clinical parameters (PD, CAL, BOP, PI, and MBL), some studies^16^^,^^17^^,^^19^^,^^20^^,^^22^^,^^24^ investigated inflammatory markers including MMPs and ILs. The findings of the majority of the included studies indicated that, in general, periodontal status was significantly worse in cigarette smokers as than in vapers or non-smokers.^18^^,^^22^^,^^24^^,^^26^

### Quality assessment of the included studies

The NOS scores obtained ranged from 3 to 6 as shown in Table 2. Nine studies were deemed of medium quality (4-6 stars) while only 1 was of low quality. Specifically, the Comparability domain yielded 1 star for only 3 studies while the remaining studies scored zero for the same domain. For the Selection and Exposure/Outcome domains, the scores ranged from 1 to 3 stars per study.

**Table 2:** Newcastle–Ottawa scale (NOS) quality assessment results.

Study	Selection	Comparability	Exposure/Outcome	Overall Star Rating	Interpretation
Akram et al.^18^	*		***	4	Medium
Al-Hamoudi et al.^19^	*		**	3	Low
Alharthi et al.^20^	*	*	***	5	Medium
Ali et al.^21^	**		**	4	Medium
ArRejaie et al.^22^	**	*	**	5	Medium
Shah et al.^23^	***		**	5	Medium
AlJasser et al.^24^	**		**	4	Medium
Alqahtani et al.^25^	**	*	**	5	Medium
BinShabaib et al.^26^	**		**	4	Medium
Ibraheem et al.^27^	*	*	**	4	Medium

A star system is used to allow a semiquantitative assessment of study quality. A study can be awarded a maximum of 1 star for each numbered item within the selection and exposure categories. A maximum of 2 stars can be given for comparability. The NOS ranges from 0 to 9/10 stars. We considered high-quality studies those that achieve ≥7, medium-quality studies 4 to 6 stars, and poor-quality study <4 stars.

For case-control studies, the selection domain assessment included whether the case definition was adequate, the representativeness of the cases, the selection of controls, and the definition of controls. Comparability was assessed on the basis of the design or analysis and whether confounders were adjusted for the analysis. The exposure domain was assessed based on ascertainment of exposure and non-response rate. For cohort studies, the selection domain was assessed based on the representativeness of the exposed cohort, selection of the non-exposed cohort, the ascertainment of exposure, and demonstration that the outcome of interest was not present at the start of the study. Comparability was assessed on the basis of the design or analysis and whether confounders were adjusted for the analysis. The outcome domain was scored based on adequate assessment of the outcome, whether follow-up was long enough for the outcome to occur, and the adequacy of follow-up of the cohort.^28^^,^^29^

### Network meta-analysis

#### Plaque index (PI)

**Fig. 2:**
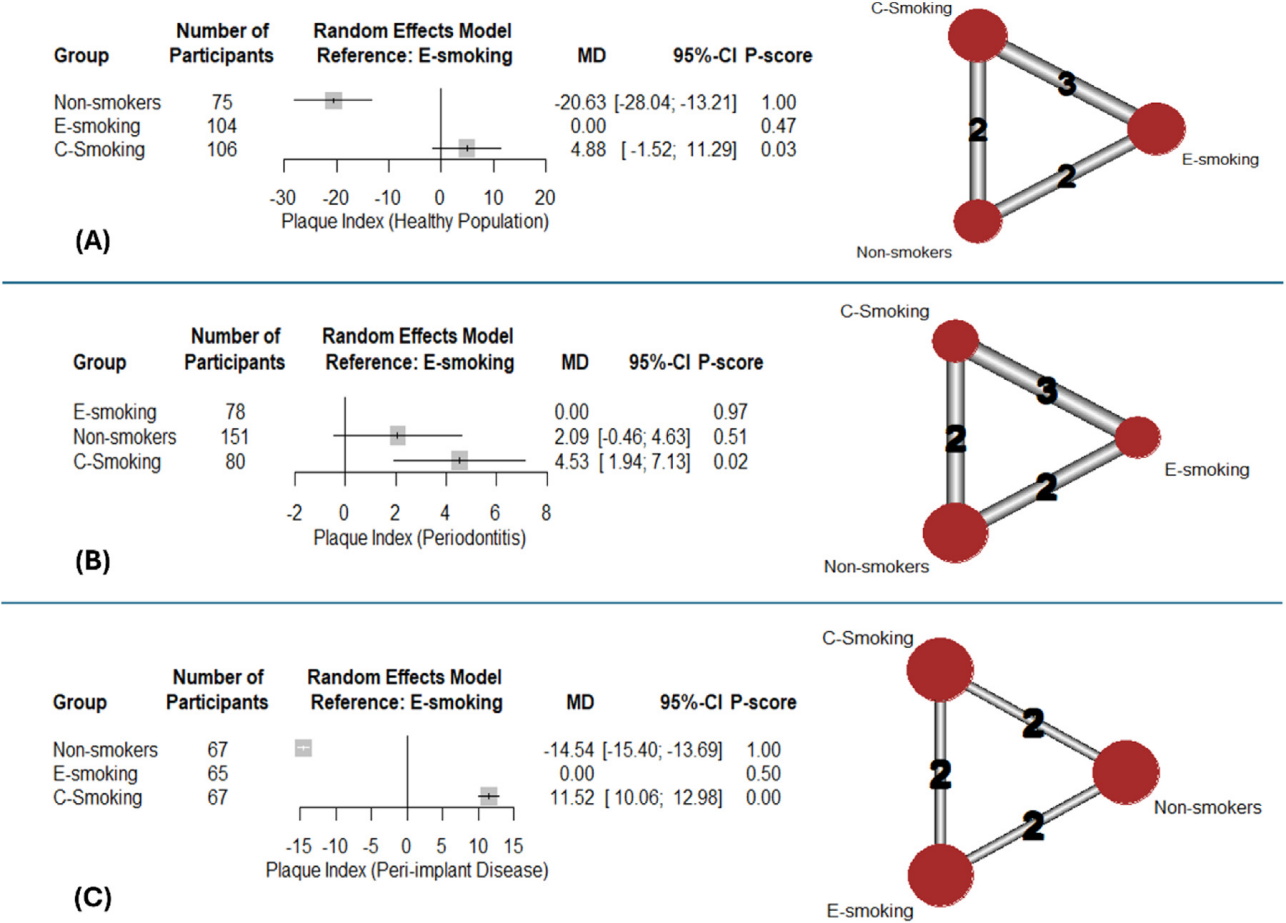
Network meta-analysis – plaque index: Forest plots with corresponding network graphs.

The NMA of healthy population of 3 studies showed that non-smokers had a significantly lower PI (MD: -20.63; 95%CI: [-28.04:-13.21]; p-value < .001) than e-smokers, while c-smoking showed slightly greater PI (MD: 4.88; 95% CI:[-1.52:11.29]; p-value = .135) (Figure 2A). Group ranking using the P-score was as follows: non-smokers (1.00), e-smokers (0.47), and c-smokers (0.03). Marked heterogeneity (I^2^=98.2%) was noted.

Among participants with periodontitis, only c-smoking had significantly higher PI (MD: 4.53; 95%CI: [1.94:7.13]; p-value < .001) (Figure 2B). According to the P-score ranking, e-smokers (0.97) had higher probabilities of better PI, followed by non-smokers (0.51), and c-smokers (0.02). No heterogeneity (I^2^=0%) was detected.

The pooled analysis of 2 studies addressing PI among patients with peri-implant disease showed it was significantly lower in non-smokers (MD: -14.54; 95%CI: [-15.40:-13.69], p-value < .001) while significantly greater PI in c-smokers was observed (MD: 11.52; 95%CI:[10.06:12:98]; p-value < .001) (Figure 2C). The rankings were non-smoker group (1), e-smoking (0.5), and c-smoking (0.0). No heterogeneity (I^2^=0%) was noted. The results of pairwise comparisons are shown in supplementary data (Table S3).

#### Bleeding on probing (BOP)

**Fig. 3:**
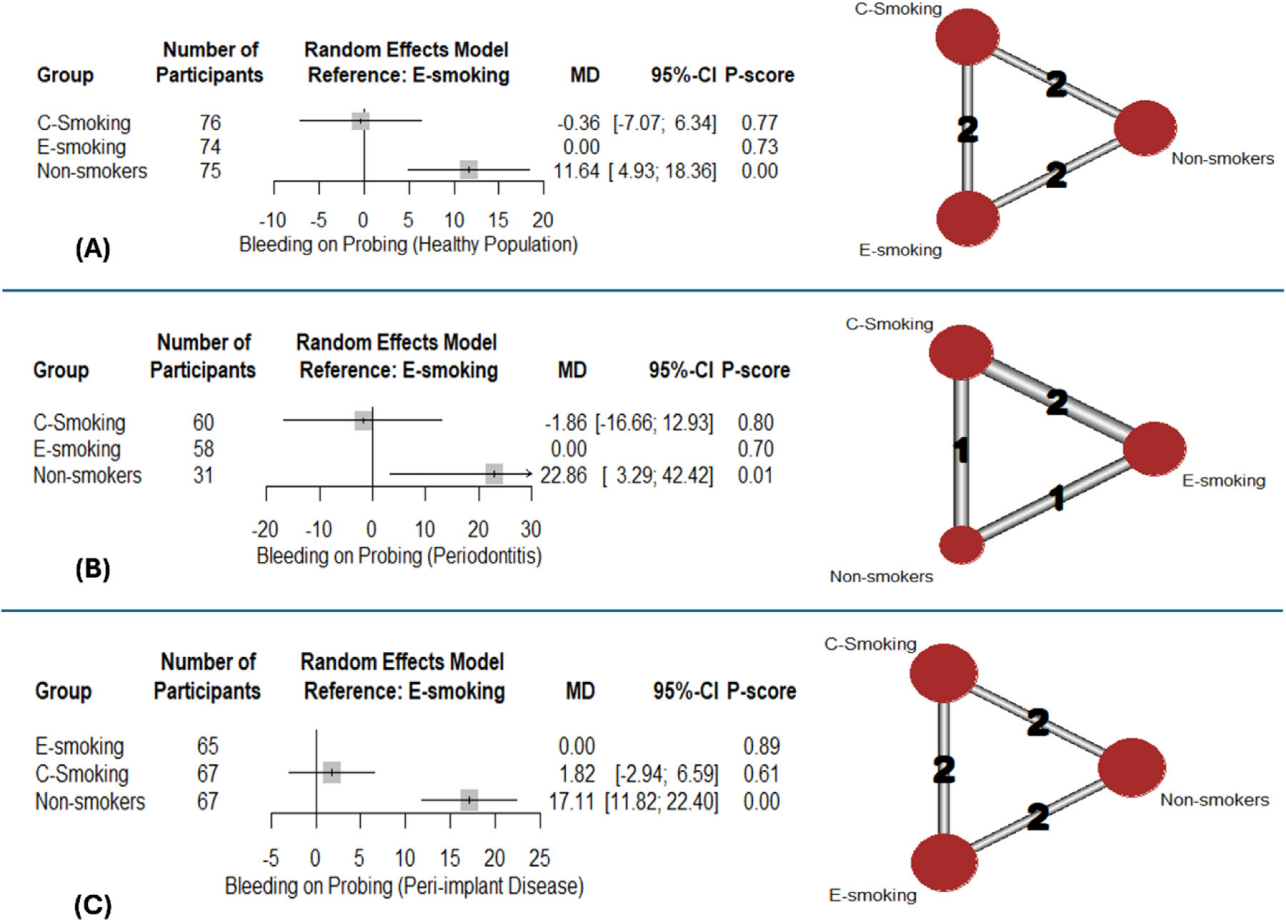
Network meta-analysis – bleeding on probing: Forest plots with corresponding network graphs.

The pooled analysis of 2 studies addressing BOP among healthy populations indicated it was significantly higher in non-smokers (MD:11.64; 95%CI: [4.93:18.36], p-value < .001) and an slightly lower in c-smokers (MD: -0.36; 95%CI: [-7.07:6.34], p-value = .91) (Figure 3A). The rankings were c-smoking (0.77), e-smoking (0.73), and lastly non-smokers (0.0). Marked heterogeneity (I^2^=99%) was noted.

Similarly, among participants with periodontitis, BOP was significantly higher among non-smokers (MD:22.86: 95%CI: [3.29:42.42], p-value = .02) (Figure 3B). Marked heterogeneity (I^2^=97%) was noted. As for patients with peri-implant disease, non-smokers also had significantly higher BOP values (MD:17.11: 95%CI: [11.82:22.40], p-value < .001) (Figure 3C). Evidence of substantial heterogeneity (I^2^=87%) was noted. The results of pairwise comparisons are shown in supplementary data (Table S4).

#### Probing depth (PD)

**Fig. 4:**
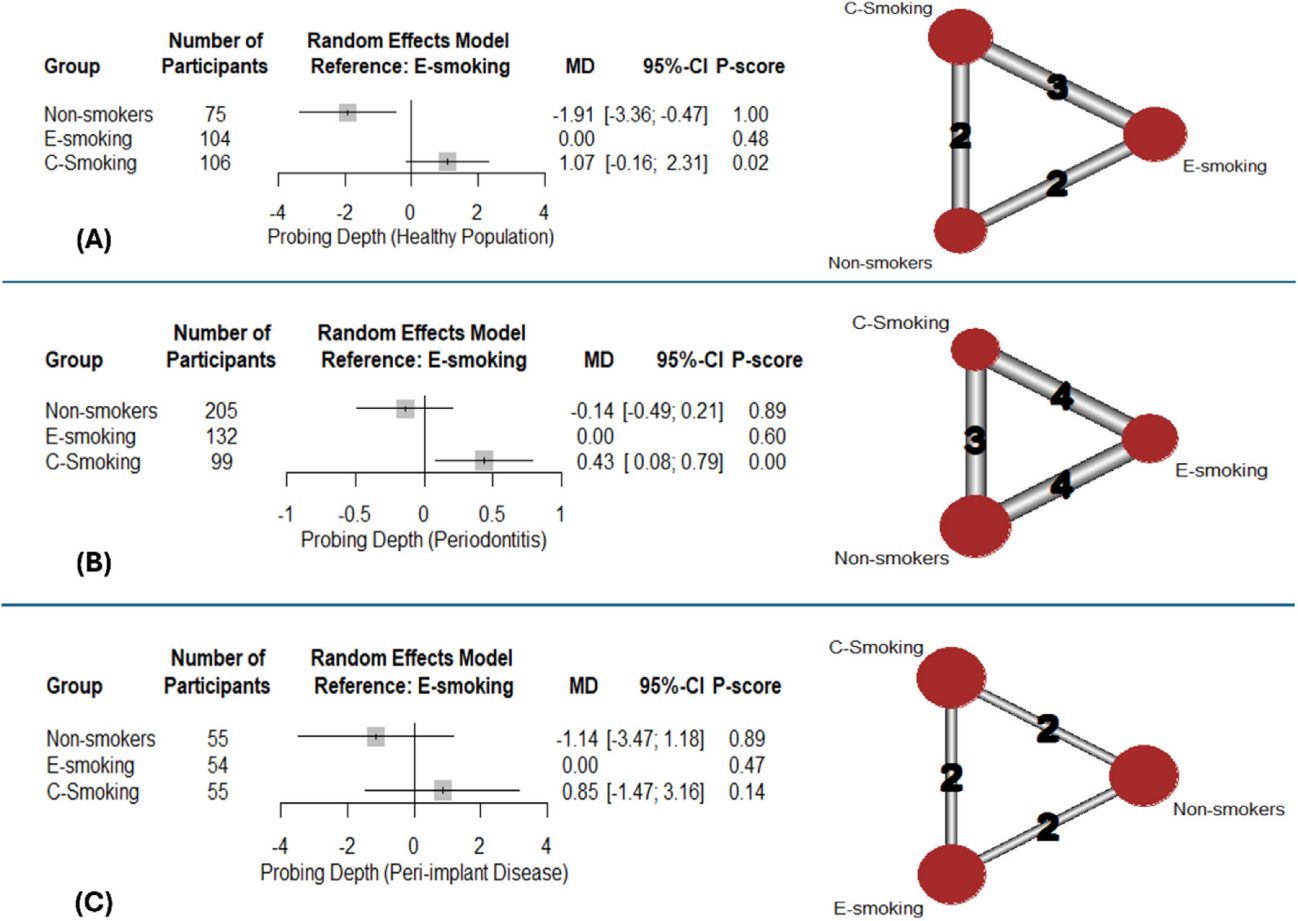
Network meta-analysis – probing depth: Forest plots with corresponding network graphs.

Pooling results of 3 studies with healthy populations showed significantly lower PD in non-smokers than e-smokers (MD: -1.91; 95% CI: [-3.36: -0.47], p-value = .01) (Figure 4A). The rankings were non-smokers (1), e-smoking (0.48), and lastly c-smoking (0.02). Marked heterogeneity (I^2^=99%) was noted.

On the other hand, pooling results of 5 studies showed that, PD was greater with c-smoking among participants with periodontitis (MD:0.43; 95%CI: [0.08:0.79]; p-value = .02) (Figure 4B). The rankings were non-smokers (0.89), e-smoking (0.60), and lastly c-smoking (0.00). Marked heterogeneity (I^2^=91%) was noted. Among participants with peri-implant disease, no significant differences were detected with either group (Figure 4C), along with the marked heterogeneity (I^2^=97%) that was noted. The results of pairwise comparisons are shown in supplementary data (Table S5).

#### Clinical attachment loss (CAL)

**Fig. 5:**
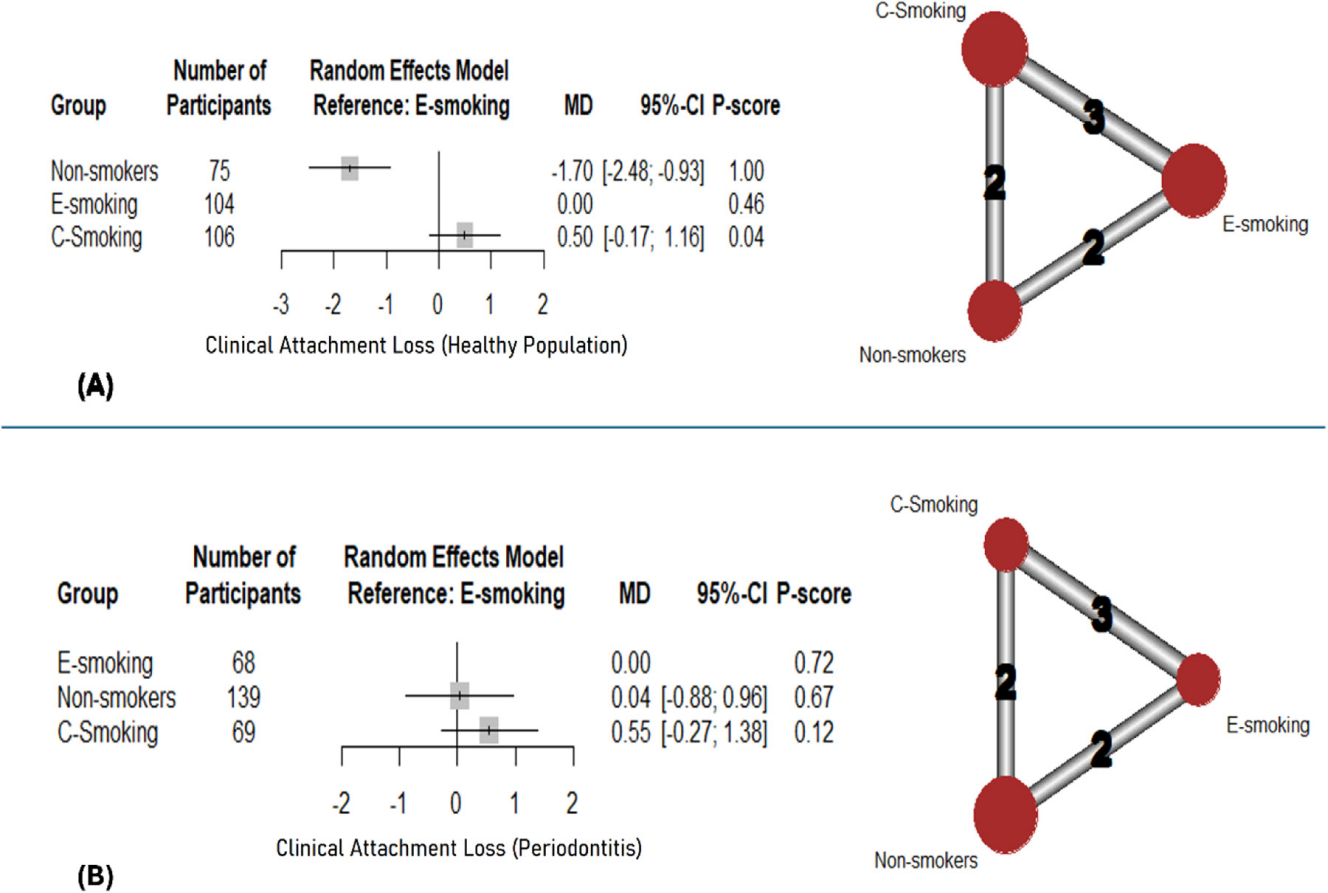
Network meta-analysis – clinical attachment loss: Forest plots with corresponding network graphs.

Pooling results of 3 studies with healthy populations showed a significantly lower CAL in non-smokers compared to e-smoking (MD: -1.70; 95%CI: [-2.48: -0.93]; p-value < .001) (Figure 5A). The rankings were non-smokers (1), e-smoking (0.46), and lastly c-smoking (0.04). Marked heterogeneity (I^2^=99%) was noted. On the other hand, on pooling results of 3 studies including participants with periodontitis, no significant differences were detected with either group (Figure 5B), along with the substantial heterogeneity (I^2^=85%) that was noted. The results of pairwise comparisons are shown in supplementary data (Table S6).

#### Total marginal bone loss (MBL)

**Fig. 6:**
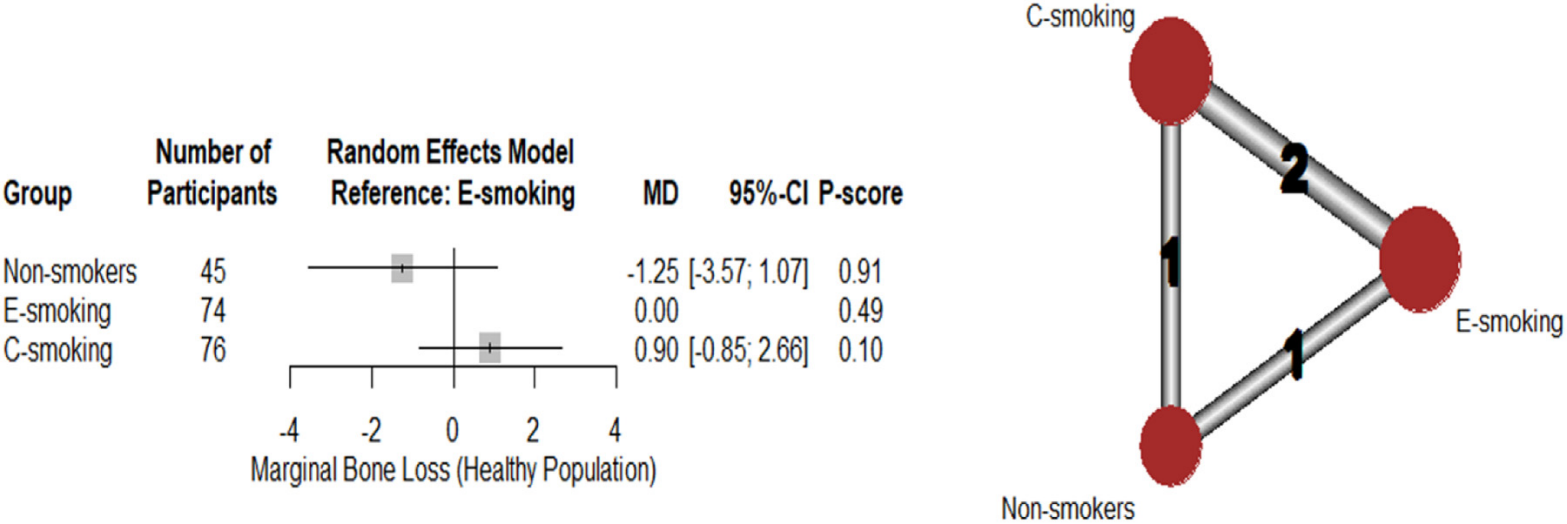
Network meta-analysis – marginal bone loss: Forest plot with corresponding network graph.

The NMA of healthy population of 2 studies showed that, compared to e-smoking, neither c-smoking (MD: 0.90; 95%CI: [-0.85:2.66], p-value = .31) nor non-smokers (MD: -1.25;95%CI: [-3.57:1.07]; p-value = .29) had significant MBL compared to e-smoking (Figure 6). Group ranking using the P-score was as follows: non-smokers (0.91), e-smoking (0.49), and c-smoking (0.1). Marked heterogeneity (I^2^=99%) was noted. The results of pairwise comparisons are shown in supplementary data (Table S7).

## Discussion

Smoking is a risk factor for several chronic diseases including periodontitis. Previous research had strongly suggested an adverse effect on the periodontal tissues. The nature of the association has not been completely uncovered. Nevertheless, it is widely accepted that smoking has both systemic and local impacts on the periodontal cells and tissues. It also affects neutrophil function and inhibits the inflammatory and immunological responses to periodontal pathogens.^32^, ^33^, ^34^, ^35^, ^36^ Reports indicated that heavy smokers have a higher incidence of periodontal pocket formation, CAL, and loss of alveolar bone, primarily explained by the fact that nicotine, along with the other toxic components of the smoke, adversely affects the host resistance to bacterial invasion caused by the plaque biofilm.^37^ Reactive aldehydes from the aerosols of e-cigs induced proteins carbonylation which is a risk factor for bone tissue injury associated with periodontitis.^38^ Other literature suggested that the evidence for the detrimental effect of nicotine, as the main risk factor, on the periodontal tissues is contradictory. A longitudinal cohort study reported that cannabis smoking, independent of the use of tobacco, was strongly associated with the prevalence and incidence of periodontal attachment loss among young adults.^39^

The recent surge in vaping has drawn attention from the healthcare and scientific community. Vaping and e-cigarette use have shown a marked increase, particularly among adolescents and young adults. This has been driven by several factors including the intense targeted marketing strategies as well as the perception that vaping might be a safer alternative to traditional smoking. Epidemiological studies have reported a significant rise in the prevalence of vaping, with some data suggesting a doubling of use rates in recent years. This trend is associated with the introduction of high-nicotine pod-based systems, which deliver nicotine more efficiently than other vaping technologies.^40^^,^^41^ Consequently, there is currently a need for a comprehensive research to explore the health implications of vaping. The aim of the current review was to summarize evidence related to the effect of vaping on the periodontal health status of e-cigarettes users.

Overall, the findings were consistent, with most of the studies suggesting that e-cigarette users are at higher risk of deteriorating periodontal health, but lower risk than conventional smokers, indicating that cigarette smoking is more harmful to the periodontal tissues, in general. Among patients with periodontitis, e-cigarettes users showed better results, in terms of PD and PI, than cigarette smokers. A possible explanation for these findings is that e-cigarette users were recovering tobacco smokers and that they still carried the periodontitis legacy from their many years of chronic tobacco smoking – and that was detectable in their periodontal status. Among participants with periodontitis, e-cigarettes users showed better results, in terms of PD and PI, than cigarette smokers. PI was significantly higher in cigarette smokers among patients with peri-implant disease as well. BinShabaib et al.^26^ compared the clinical and radiographic periodontal parameters and GCF levels of proinflammatory cytokines among cigarette smokers, and e-cigarette users. Their results indicated significantly higher mean scores of PD, PI, and CAL among cigarette smokers. Similarly, the study by Akram et al.^18^ reported a significantly higher CAL at 6 months for both cigarette smokers and e-cigarettes users however, this difference was significantly higher for cigarette smokers than in e-cigarettes users. ArRejaie et al.^22^ indicated that peri-implant health was more compromised in cigarette smokers than vapers with higher levels of proinflammatory cytokines suggesting a greater peri-implant inflammatory response. In all studies, BOP showed higher values in nonsmokers than in cigarette smokers or vapers, which could be due to the vasoconstrictive nature of nicotine and its effect on the gingival tissues.^37^

Some studies investigated the effect of smoking status on periodontal treatment response. The study by Alharthi et al.^20^ demonstrated that cigarette smokers had a poorer response, in terms of sites with PD ≥4 mm at 6 months follow-up, following full-mouth ultrasonic scaling compared to individuals vaping e-cigarettes. The study by Shah et al.^23^ reported that, compared with nonsmokers, vapers had a less favorable treatment response after mechanical plaque removal including statistically significant increased “need for surgery,” as well as an increased number of sextants with PD ≥5 mm, and mean PD however, their response to treatment was statistically non-significant compared to cigarette smokers.

It is worth noting that, according to the risk of bias assessment, the certainty of evidence and the quality of the included studies ranged from low to medium. This significantly affects the reliability of the conclusions drawn from them. Subgroup analysis was done only whenever possible due to insufficient sample sizes and lack of representation in most of the studies. Some studies had significant discrepancies in patients’ characteristics and outcome measurements. This entails the need for caution when attempting to generalize from these findings.

## Conclusion

Based on the current analysis, conventional cigarette smoking is the most detrimental to periodontal health among the groups compared in all included studies. This indicates that traditional cigarettes have a more severe impact on periodontal cells and tissues than do e-cigarettes. The data suggest a gradient of risk where nonsmokers have the lowest risk, e-cigarettes users have a moderate risk, and cigarette smokers have the highest risk for periodontal health issues. Additional research is needed to validate the evidence presented. There is clearly an urgent need for larger, more comprehensive studies that fully investigate the precise mechanism of e-cigarettes' effects on periodontal tissues.

## Conflict of interest

The authors declare that they have no known competing financial interests or personal relationships that could have appeared to influence the work reported in this paper.

## Appendix Supplementary materials

Image, application 1

## Footnotes

Supplementary material associated with this article can be found in the online version at doi:10.1016/j.identj.2024.12.036.
