# Supplementary material for: Effects of Electronic Cigarettes on Periodontal Health: A Systematic Review and Meta-Analysis
Source: Int Dent J. 2025 Jan 24;75(3):2014–24. doi: 10.1016/j.identj.2024.12.036 (PMC12142770; doi:10.1016/j.identj.2024.12.036)
Supplement: Supplementary file 1 [file mmc1.docx]

**SUPPLEMENTARY FILES**

**Table S1:** The review elements and search details.

| **Review Elements** | **Population** | Patients 16 years old and older using e- cigarettes |
| --- | --- | --- |
|  | **Intervention (Risk Factor)** | Electronic smoking |
|  | **Control** | Conventional smoking or no smoking |
|  | **Outcome** | Periodontal health parameters |
| **Inclusion Criteria** | | Observational studies fulfilling the abovementioned elements will be included. |
| **Exclusion Criteria** | | Review articles, theses, conference abstracts, editorials, commentaries, case reports, articles written in languages other than English, and in vitro or animal studies. |
| **Search Strategy** | | (“oral health” OR “dental health” OR “dental disease” OR “mouth disease” OR “oral disease” OR oral injury* OR “periodontal health” OR “periodontal disease” OR “periodontitis” OR “oral hygiene” OR “oral wound healing” OR “oral mucosa” OR “peri-implantitis” OR “peri implantitis” OR “probing index” OR “bleeding on probing” OR “plaque index” OR “attachment loss” OR “alveolar bone loss”) AND (“electronic cigarette” OR “e-cigarette” OR vape* OR “electronic nicotine delivery system” OR “Ecigarette” OR “vaping” OR “E cigarette” OR “E-cig” OR “E cig” OR “vaporizer”) |
| **Date of search** | | From inception till January 31^st,^ 2024 |

**Tables S2:** Newcastle–Ottawa Scale (NOS) quality assessment results for each study

| Study | Selection | Comparability | Exposure/Outcome |
| --- | --- | --- | --- |
| Akram et al., 2021^18^ | *(for the selection of the non-exposed cohort from the same community as the exposed cohort)  -Non representative exposed cohort  -No ascertainment of exposure | Study did not control for confounders | *** (for independent blind assessment;  Follow up ≥6 months) |
| Al-Hamoudi et al, 2020^19^ | *(for the selection of the non-exposed cohort from the same community as the exposed cohort)  -Non representative exposed cohort  -No ascertainment of exposure | Study did not control for confounders | **(for independent blind assessment;  Follow up ˂6 months) |
| Alharthi et al, 2019^20^ | *(for the selection of the non-exposed cohort from the same community as the exposed cohort)  -Non representative exposed cohort  -No ascertainment of exposure | * (The study controls for the sex (male subjects) | ***(for independent blind assessment;  Follow up ≥6 months) |
| Ali et al, 2023^21^ | ** (For sample size predetermined and calculated, measurement tool described)  -No description of the sampling strategy  -Selected group of users | Study did not control for confounders | ** (for independent blind assessment) |
| ArRejaie et al, 2019^22^ | ** (For sample size predetermined and calculated, measurement tool described)  -No description of the sampling strategy  -Selected group of users | *(The study controls for the sex (male subjects) | ** (for independent blind assessment) |
| Shah et al, 2023^23^ | ***(Ascertainment of exposure, selection of the non-exposed cohort from the same community as the exposed cohort, outcome of interest was not present at start of the study)  -Non representative exposed cohort | Study did not control for confounders | **(Independent blind assessment) |
| AlJasser et al, 2022^24^ | **(Ascertainment of exposure, selection of the non-exposed cohort from the same community as the exposed cohort)  -Non representative exposed cohort | Study did not control for confounders | **(Independent blind assessment) |
| Alqahtani et al, 2019^25^ | ** (For sample size predetermined and calculated, measurement tool described)  -No description of the sampling strategy  -Selected group of users | The study controls for the sex (male subjects) | ** (for independent blind assessment) |
| BinShabaib et al, 2019^26^ | ** (for sample size predetermined and calculated, measurement tool described)  -No description of the sampling strategy  -Selected group of users | Study did not control for confounders | **(for record linkage, clinical and radiographic findings) |
| Ibraheem et al, 2020^27^ | *(for sample size predetermined and calculated)  -No description of the sampling strategy or measurement tool  -Selected group of users | The study controls for the sex (male subjects) | **(for record linkage, clinical and radiographic findings) |

**Tables S3:S7** represent the league tables of the included outcomes.

Results of the network meta-analysis are presented in the left lower half and results from pairwise meta-analysis in the upper right half, if available. The effect estimate is described as mean difference (MD) with the 95% confidence interval (CI). In the lower left half, an MD below 0 favors the column-defining treatment (less severe) while on the upper right half, an MD below 0 favors the row-defining treatment (less severe). The significant values are in bold.

**Table S3**: League table of the plaque index (PI) outcome of different population subgroups.

| **Plaque Index** | | |
| --- | --- | --- |
| **Healthy Population** | | |
| **C-Smoking** | 4.91 ( -1.50 to 11.31) | **26.07 ( 18.29 to 33.84)** |
| 4.88 ( -1.52 to 11.29) | **E-smoking** | **20.05 ( 12.32 to 27.77)** |
| **25.51 ( 18.06 to 32.96)** | **20.63 ( 13.21 to 28.04)** | **Non-smokers** |
| **Periodontitis Patients** | | |
| **C-Smoking** | **4.51 ( 1.91 to 7.11)** | 2.95 (-0.09 to 5.99) |
| **4.53 ( 1.94 to 7.13)** | **E-smoking** | -2.44 (-5.10 to 0.23) |
| 2.45 (-0.36 to 5.25) | -2.09 (-4.63 to 0.46) | **Non-smokers** |
| **Peri-implant Disease Patients** | | |
| **C-Smoking** | **11.52 ( 10.06 to 12.98)** | **26.05 ( 24.77 to 27.34)** |
| **11.52 ( 10.06 to 12.98)** | **E-smoking** | **14.55 ( 13.69 to 15.40)** |
| **26.06 ( 24.78 to 27.35)** | **14.54 ( 13.69 to 15.40)** | **Non-smokers** |

**Table S4**: League table of the bleeding on probing (BOP) outcome of different population subgroups.

| **Bleeding on Probing** | | |
| --- | --- | --- |
| **Healthy Population** | | |
| **C-Smoking** | -0.35 ( -7.06 to 6.35) | **-12.00 (-18.73 to -5.27)** |
| -0.36 ( -7.07 to 6.34) | **E-smoking** | **-11.63 (-18.34 to -4.91)** |
| **-12.01 (-18.74 to -5.28)** | **-11.64 (-18.36 to -4.93)** | **Non-smokers** |
| **Periodontitis Patients** | | |
| **C-Smoking** | -1.86 (-16.66 to 12.93) | **-21.00 (-41.86 to -0.14)** |
| -1.86 (-16.66 to12.93) | **E-smoking** | **-26.60 (-47.50 to -5.70)** |
| **-24.72 (-44.27 to -5.17)** | **-22.86 (-42.42 to -3.29)** | **Non-smokers** |
| **Peri-implant Disease Patients** | | |
| **C-Smoking** | 1.84 ( -2.93 to 6.60) | **-15.82 (-21.14 to -10.50)** |
| 1.82 ( -2.94 to 6.59) | **E-smoking** | **-17.17 (-22.51 to -11.84)** |
| **-15.29 (-20.57 to -10.01)** | **-17.11 (-22.40 to -11.82)** | **Non-smokers** |

**Table S5**: League table of the probing depth (PD) outcome of different population subgroups.

| **Probing Depth** | | |
| --- | --- | --- |
| **Healthy Population** | | |
| **C-Smoking** | 1.07 (-0.16 to 2.31) | **3.25 ( 1.74 to 4.76)** |
| 1.07 (-0.16 to 2.31) | **E-smoking** | **1.65 ( 0.14 to 3.16)** |
| **2.99 ( 1.54 to 4.43)** | **1.91 ( 0.47 to 3.36)** | **Non-smokers** |
| **Periodontitis Patients** | | |
| **C-Smoking** | **0.44 ( 0.08 to 0.80)** | **0.59 ( 0.17 to 1.01)** |
| **0.43 ( 0.08 to 0.79)** | **E-smoking** | 0.12 (-0.24 to 0.48) |
| **0.57 ( 0.19 to 0.96)** | 0.14 (-0.21 to 0.49) | **Non-smokers** |
| **Peri-implant Disease Patients** | | |
| **C-Smoking** | 0.86 (-1.46 to 3.17) | 1.98 (-0.33 to 4.30) |
| 0.85 (-1.47 to 3.16) | **E-smoking** | 1.14 (-1.18 to 3.47) |
| 1.99 (-0.33 to 4.30) | 1.14 (-1.18 to 3.47) | **Non-smokers** |

**Table S6**: League table of the attachment loss (AL) outcome of different population subgroups.

| **Attachment Loss** | | |
| --- | --- | --- |
| **Healthy Population** | | |
| **C-Smoking** | 0.50 (-0.16 to 1.16) | **2.30 ( 1.49 to 3.11)** |
| 0.50 (-0.17 to 1.16) | **E-smoking** | **1.60 ( 0.79 to 2.41)** |
| **2.20 ( 1.42 to 2.97)** | **1.70 ( 0.93 to 2.48)** | **Non-smokers** |
| **Periodontitis** | | |
| **C-Smoking** | 0.55 (-0.27 to 1.37) | 0.53 (-0.43 to 1.49) |
| 0.55 (-0.27 to 1.38) | **E-smoking** | -0.07 (-1.03 to 0.89) |
| 0.51 (-0.41 to 1.43) | -0.04 (-0.96 to 0.88) | **Non-smokers** |

**Table S7**: League table of the total marginal bone loss (MBL) outcome.

| **Total Marginal Bone Loss** | | |
| --- | --- | --- |
| **Healthy Population** | | |
| **C-smoking** | 0.90 (-0.85 to 2.66) | **2.60 ( 0.12 to 5.08)** |
| 0.90 (-0.85 to 2.66) | **E-smoking** | 0.80 (-1.68 to 3.28) |
| 2.15 (-0.17 to 4.47) | 1.25 (-1.07 to 3.57) | **Non-smokers** |
